# Self-help psychological intervention for young individuals during the post-COVID-19 era: development of a PST chatbot using GPT-4

**DOI:** 10.3389/fdgth.2025.1627268

**Published:** 2025-09-23

**Authors:** Liuling Mo, He Li, Yanbo Zhang, Ang Li, Ziyue Xiong, Peixin Cun, Tingshao Zhu

**Affiliations:** Department of Social Psychology, School of Sociology, Nankai University, Tianjin, China

**Keywords:** post-COVID-19 era, young individuals, self-help psychological intervention, GPT-4, problem-solving therapy, chatbot

## Abstract

**Introduction:**

The COVID-19 pandemic has exacerbated psychological stress among young people, with some survivors experiencing persistent mental distress, thus creating an urgent need for accessible psychological intervention tools. To help young people affected by COVID-19 recover and achieve balanced mental health in the post-pandemic era, this study developed an online self-help psychological intervention chatbot to supplement existing mental health resources.

**Methods:**

We utilized prompt engineering techniques to construct a chatbot proficient in Problem-Solving Therapy (PST) based on the large language model GPT-4. Subsequently, 7 master's students majoring in psychological counseling were recruited for a pre-test of the chatbot, and 100 young people who had contracted COVID-19 were selected for a formal user experiment to evaluate its effectiveness.

**Results:**

The pre-test results indicated that the chatbot followed the core steps of PST during interactions with users and was helpful in problem-solving. The formal experiment showed that the experimental group scored significantly higher than the control group in the dimensions of problem awareness [*t* (88.31) = 3.14, *p* = 0.002] and problem-solving [*t* (98) = 3.34, *p* = 0.001], but there was no significant difference between the two groups in the dimension of relationship quality [*t* (91.23) = 1.07, *p* = 0.286]. In addition, no significant differences were found in the evaluation based on gender or the presence of post-COVID-19 symptoms, indicating that the chatbot has a certain degree of universal applicability.

**Conclusions:**

These findings support the application of the PST chatbot in post-COVID-19 era psychological interventions, particularly in assisting users with identifying problems and exploring solutions. Although the chatbot did not achieve significant improvement in human-computer relationship quality, its general acceptability and broad applicability demonstrate great potential in the field of mental health, highlighting the value of large language models in promoting self-help mental health interventions as a supplementary tool to existing resources.

## 1 Introduction

The COVID-19 pandemic has significantly exacerbated psychological stress among young individuals due to prolonged uncertainty, social isolation, and disruptions to daily life, exerting sustained impacts on their mental health. As of December 17, 2023, approximately 773 million confirmed cases of COVID-19 have been reported globally (1), with many young survivors continuing to suffer from mental health issues such as anxiety, depression, post-traumatic stress disorder, and cognitive impairments (2–4). These problems often manifest as reduced problem-solving abilities, heightened feelings of powerlessness, and increased suicide risk (5), highlighting an urgent need for accessible mental health support.

Social support is widely recognized as a critical protective factor against such risks. Studies have emphasized that robust social support networks can provide emotional comfort, informational guidance, and practical assistance (6), while others have found that individuals who perceive care and support are more likely to seek help during crises (7). However, traditional mental health resources face multiple limitations in the post-pandemic era, including shortages of professionals, high costs, and social stigma associated with seeking face-to-face therapy (8). This gap underscores the urgent need for scalable, low-cost interventions to supplement existing services.

Digital mental health tools, particularly chatbots, have emerged as promising solutions. They offer 24/7 accessibility, anonymity, and consistent interventions without the resource constraints of human-led therapy (9). For instance, research has shown that generative chatbots can effectively deliver psychological support (10), and other studies have highlighted the potential of empathetic design in enhancing user engagement with mental health chatbots (11). Despite these advancements, few studies have integrated evidence-based therapeutic frameworks into chatbots to specifically address the post-COVID mental health issues of young people.

This study addresses this gap by developing a chatbot based on Problem-Solving Therapy (PST). PST is a well-established psychotherapeutic approach that improves mental health by enhancing individuals' abilities to resolve daily life problems (12, 13). Like Cognitive Behavioral Therapy (CBT), PST falls under the category of “action-oriented” therapies, as both emphasize alleviating distress through modifying cognition and behavior (14). However, PST places greater emphasis on the practical resolution of specific problems and involves a more streamlined exploration of cognitive restructuring compared to CBT. In contrast to supportive therapy (15), PST not only provides emotional support but also focuses on empowering users with the capacity to solve problems independently.

The core premise of PST—that problem-solving skills directly influence mental health—aligns with the unique needs of young people, who often lack extensive life experience and may experience cognitive decline as a post-COVID sequela (3), thereby hindering their problem-solving capacities. PST systematically guides users through problem identification, goal-setting, solution exploration, and action planning, thereby enhancing self-efficacy and reducing distress (13). Previous work by our team has demonstrated the effectiveness of PST in large-scale interventions: during a 2016–2020 Weibo-based project, volunteers provided PST-informed support to 3,627 users, accumulating over 127,000 interactions with positive feedback (16). However, the project stalled during the pandemic due to resource demands and volunteer burnout. Chatbots can overcome these limitations by delivering consistent PST interventions at scale (9).

Leveraging recent advancements in large language models (LLMs) and prompt engineering (PE), we aim to operationalize PST within a chatbot. Research has highlighted that PE is a critical tool for aligning LLMs with therapeutic goals, ensuring interventions adhere to evidence-based protocols (17). By integrating PST into a GPT-4-powered chatbot via PE, we seek to create a self-help tool that assists young post-COVID survivors in identifying problems, exploring solutions, and rebuilding psychological resilience.

In summary, the objectives of this study are: (1) to develop a PST-informed chatbot using GPT-4 and PE; (2) to evaluate its effectiveness in enhancing young COVID survivors' abilities to recognize and solve problems; (3) to assess its acceptability among populations with different genders and post-COVID symptom statuses. This work contributes to the growing field of digital mental health research by demonstrating how LLMs can be used to deliver structured, scalable interventions, ultimately serving as an important supplement to traditional mental health resources.

## 2 Methods

### 2.1 Step 1: construction and preliminary testing of the PST chatbot

#### 2.1.1 Participants

During the preliminary testing phase, 7 master's degree students specializing in psychological counseling were recruited. All were volunteers who had received training in Problem-Solving Therapy as part of the Weibo online rescue project, and they evaluated the PST chatbot.

#### 2.1.2 Experimental procedure

##### 2.1.2.1 Creating prompts to guide GPT-4 outputs

Through Large Language Models (LLMs) and Prompt Engineering (PE), we can integrate Problem-Solving Therapy into chatbot programs to assist individuals in managing their issues, thereby enhancing the accessibility and efficiency of psychological interventions. Large Language Models, such as GPT-3 or GPT-4, are artificial intelligence models based on deep learning that are capable of understanding and generating human language. These models learn the intricacies of language and how to respond to questions, provide information, and compose texts by training on vast amounts of text data.

PE is an emerging concept in the field of artificial intelligence, particularly within the subdomains of Natural Language Processing (NLP) and machine learning. It involves the creation and refinement of carefully designed prompts to guide large language models to produce specific, anticipated outputs. This technique is especially important for models based on the Transformer architecture, such as the Generative Pre-trained Transformer (GPT) series, as these models typically require text generation based on given contexts. The Transformer architecture has propelled the development of artificial intelligence technology and serves as the fundamental architecture for numerous advanced language models and deep learning models. With the self-attention mechanism at its core, it possesses the capability of parallel computing. Comprising an encoder and a decoder, it can encode inputs and generate outputs (18). Effective prompts can significantly enhance a model's performance on specific tasks, sometimes even reaching or surpassing the level of specially trained models (19). Therefore, the rational and systematic guidance framework of PST and the extensive knowledge base of LLMs can provide strong support to the youth in the post-pandemic era. These resources enable young people to better cope with the challenges they face, enhance their problem-solving skills, and promote their mental health.

**Figure 1:**
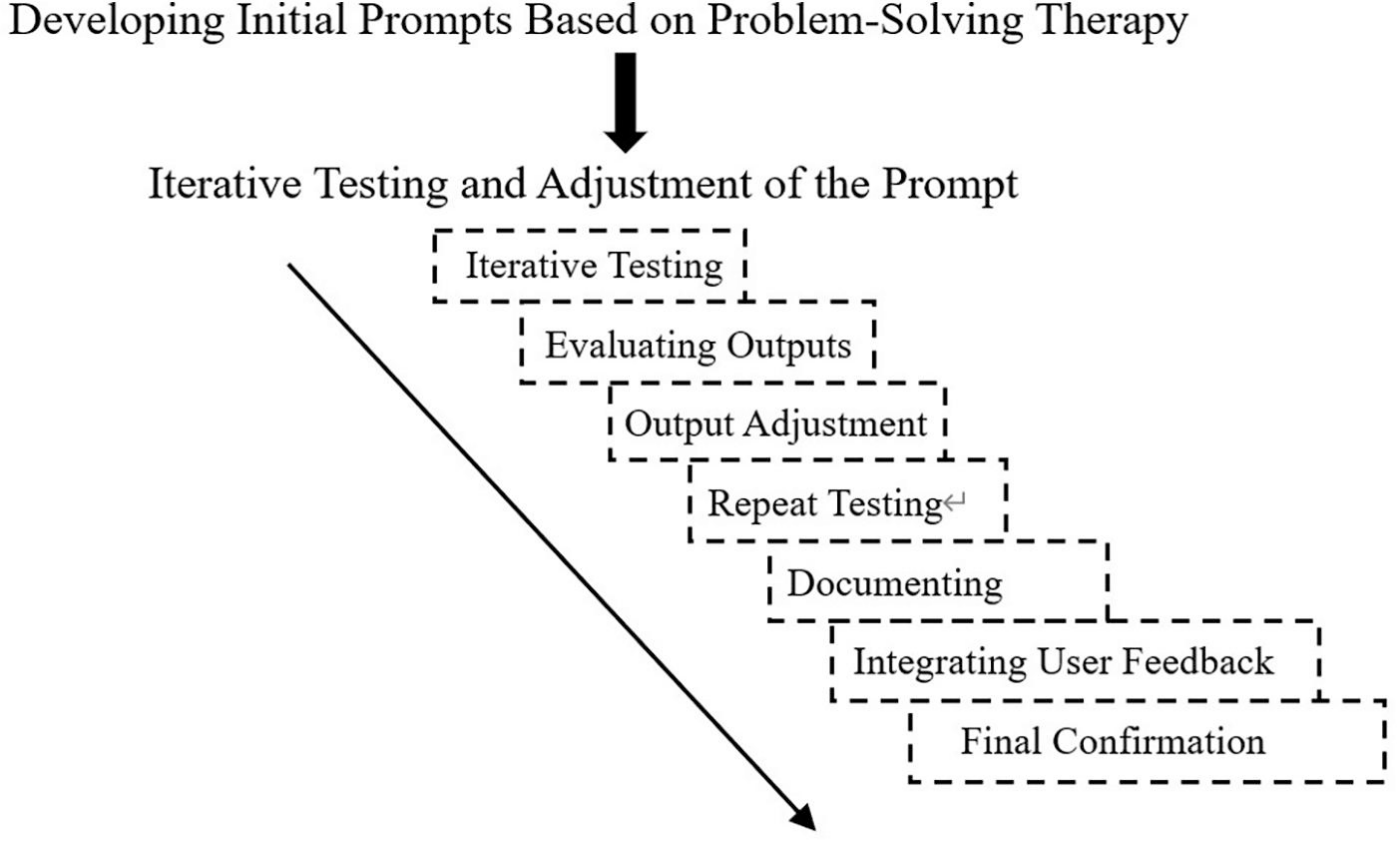
Prompt design process.

Using PE techniques, appropriate prompts guide the Large Language Model GPT-4 to generate outputs aligned with the principles of Problem-Solving Therapy. Firstly, prompts are created to ensure that the chatbot's dialogue logic adheres to the core principles and procedures of Problem-Solving Therapy (PST). These designed prompts are then used to interact with the model, and the consistency of the outputs with the expected results is observed. Based on the test outcomes, the structure, language, and details of the prompts are adjusted to refine the model's outputs. After several rounds of iterative testing, the final suitable prompts are determined. The specific process is illustrated in Figure 1. For more information on the application of prompt engineering in the field of mental health, please refer to the review article published by Priyadarshana et al. in 2024 (17).

Initial Prompt Writing: When constructing the initial prompt within the context of Problem-Solving Therapy (PST), initially, a researcher proficient in prompt engineering provided a fundamental framework for prompt word composition, namely: Role-Description-Skills-Workflow-Rules-Initialization. Subsequently, a psychological counselor skilled in PST designed the initial prompt in accordance with this framework. Additionally, iterative testing and adjustment were carried out on the initial prompt to direct GPT4 to offer assistance centered around the core steps of problem-solving therapy.

Iterative Testing: The designed initial prompt was used to guide the large-language model. The initial interaction might be a simple user input like: “I've been feeling a bit unhappy lately and my life is in a state of confusion.” Then, we observed whether the model's response could prompt the user to disclose the real problems that were bothering them, provide goal-setting methods consistent with PST, guide the user to explore possible solutions, and ultimately help the user formulate an action plan.

Evaluating Outputs: Confirm whether the model output follows the logic of PST. Firstly, it should be problem identification, followed by goal-setting, then exploration of solutions, and finally the formulation of an action plan. Evaluate the quality of the content, including the practicality of suggestions, respect for user autonomy, and determine whether it helps users think about problems from different perspectives.

Output Adjustment: If the model's response fails to correctly follow the steps of PST or the provided solutions are unrealistic, then the prompt needs to be adjusted, for example, by increasing the clarity of the prompt.

Repeat testing: Retest using the adjusted prompt, collect new outputs, and then evaluate again. This process may require multiple iterations, with each iteration making fine-tuned adjustments based on the observed problems.

Documenting: After each iteration, record which adjustments were effective and which were not. Documentation helps in understanding what types of prompts can generate outputs closer to expectations, and also facilitates the faster optimization of prompts for similar tasks in the future.

Integrating User Feedback (Pre-test): Recruit master's degree students majoring in psychology counseling who have received training in problem-solving therapy to try out and evaluate the PST chatbot as users. Determine whether the responses of the PST robot are truly easy for users to understand and whether they meet the actual needs and feelings of users.

Final Confirmation: After multiple tests, adjustments, and evaluations, an optimized prompt is obtained. This prompt helps the chatbot efficiently assist users in handling and solving user problems according to the PST method.

##### 2.1.2.2 Setting up a PST chatbot website

**Figure 2:**
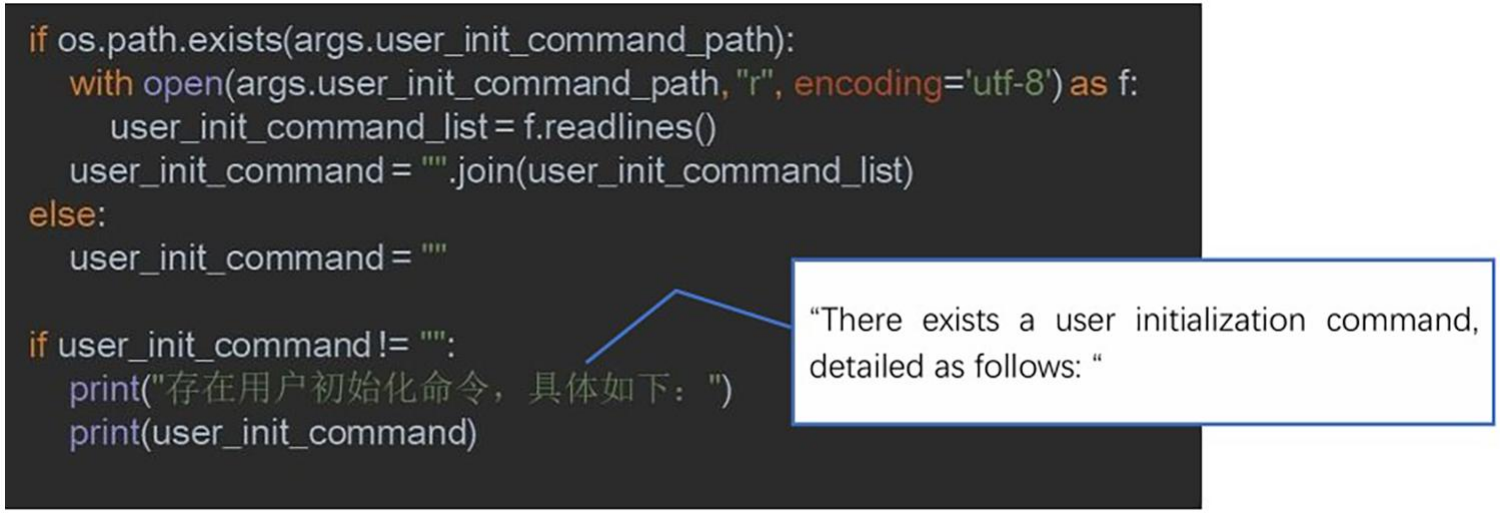
Code example for initializing a chatbot using a pre-trained prompt.

Developing a Self-Help Psychological Intervention Chatbot Program in Python involves several key functionalities. They include invoking GPT-4 to create a self-help psychological intervention chatbot, initializing the chatbot with a pre-trained prompt (see example code in Figure 2), and using Gradio to construct a website for user interaction. Gradio is an open-source Python library that simplifies building user interfaces for machine learning models. With it, developers can create professional-level interfaces in just a few lines of code. The interface provides a clean, user-friendly default layout and allows customization of theme colors, font styles, and other design elements. The final PST chatbot operates according to the four core steps of Problem-Solving Therapy (13):

1. Problem Identification: The chatbot identifies the visitor's primary concerns and challenges. It encourages users to express their troubles or issues directly to the chatbot, ensuring that the user feels understood and clarifying the specific problem. For example, “How has this problem impacted your life?”
2. Goal Setting: Clear goal setting is crucial for successfully resolving issues. The chatbot guides users to consider what their ideal outcome would be and helps them break down this goal into smaller, more specific action items. For example, “What changes do you hope to see in your life or emotional state after resolving this problem?”
3. Exploring Solutions: The chatbot encourage users to think broadly and consider various possible solutions. It offers guiding questions to foster thought, such as, “Can you think of any strategies that have helped you deal with similar issues before?” Additionally, the chatbot can provide suggestions and resources as needed.
4. Making a Plan: The chatbot supports the user in devising specific action steps, increasing the user's sense of efficacy in solving the problem. This may include determining a timeline, identifying resources, seeking interpersonal support, and anticipating and planning for potential difficulties. For example, “Let's determine your first step of action and when you will start.”

##### 2.1.2.3 Pre-test

**Figure 3:**
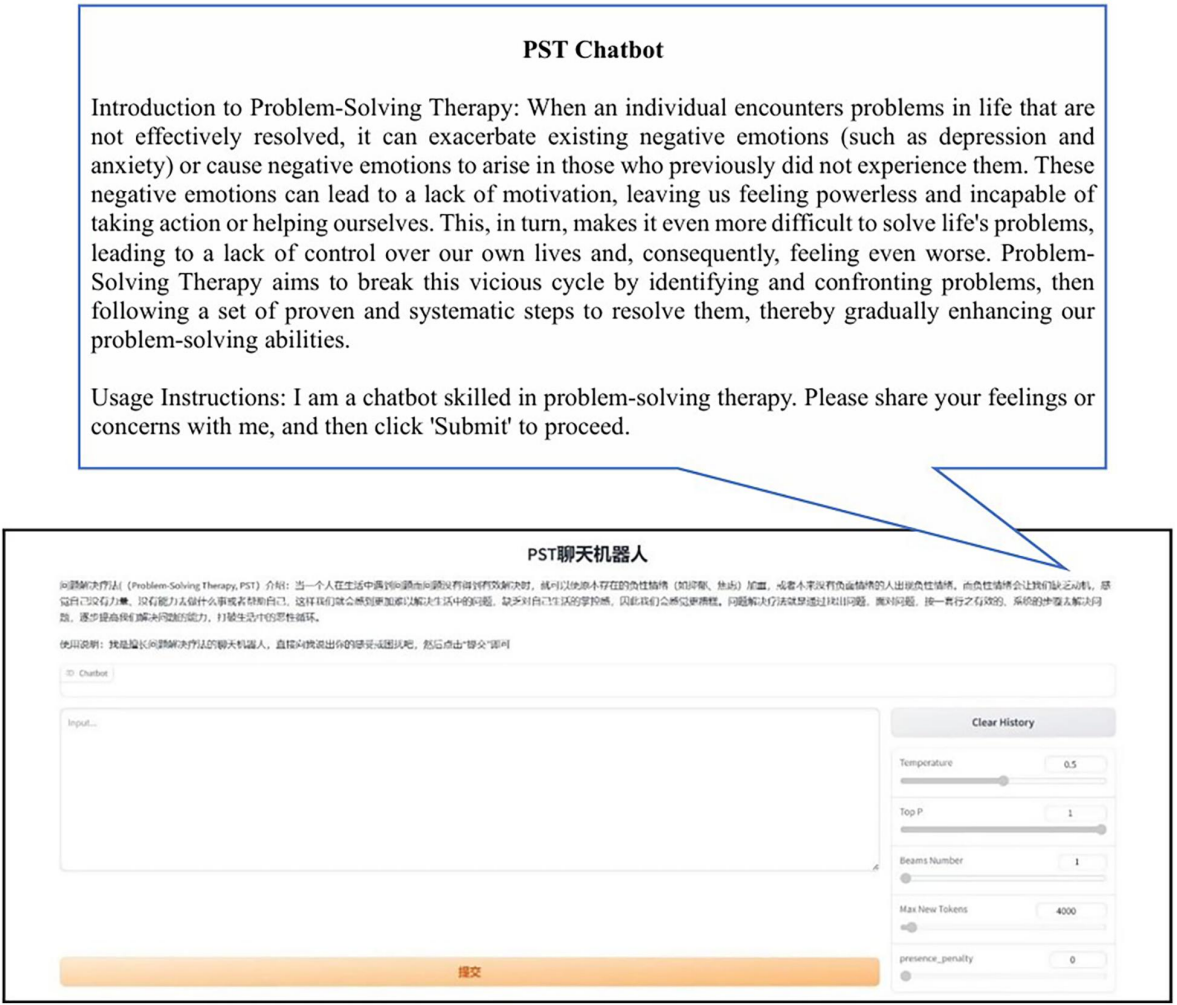
Pre-test chatbot Web interface instruction page.

In this research, we carried out the following pre-test. We introduced the functionality, intended use, and specific rating criteria of the PST chatbot in detail to 7 volunteers, and had them interact with the PST chatbot. The instructions for the pre-test webpage can be found in Figure 3. The principal investigator provided immediate technical support if the volunteers encounter any issues. Encourage them to interact with the chatbot naturally, as they would with a real person in a real-life situation.

After the test concluded, volunteer feedback was collected using a questionnaire format. This is carried out immediately after the conversation to ensure the accuracy of the feedback. The feedback should include: a. Whether the PST chatbot adheres to the process of problem-solving therapy; b. Whether the PST chatbot is helpful in solving the problems that trouble them; c. Specific suggestions for improvements to the PST chatbot; d. The duration of a complete chat session. The specific content of the questionnaire is shown in Table 1.

**Table 1:** Volunteer feedback content.

Feedback content	Description
a. Degree of adherence to the Problem-Solving Therapy (PST) process:	Rate from 1 to 10, with higher scores indicating strong adherence to the PST protocol, demonstrating the extent to which the chatbot follows or deviates from the PST process.
b. Level of assistance in resolving distress	Rate from 1 to 10, with higher scores indicating that the PST chatbot is more effective in helping users with their issues.
c. Specific suggestions for improvement	Invite volunteers to offer concrete suggestions for enhancements based on their professional knowledge and interactive experience.
d. Total chat duration	Record the total length of interaction between the volunteer and the chatbot to gauge the volunteer's level of engagement and the time efficiency of each session.

### 2.2 Step 2: formal user experiment: a randomized controlled trial

#### 2.2.1 Study design

Randomized Controlled Trial (RCT) is the gold standard for evaluating the effectiveness of mental health chatbots, as it can effectively control confounding variables and ensure causal inference of results (20, 21). Additionally, by referring to previous RCT studies on mental health chatbots, we consider that recruiting approximately 100 participants for the experiment will suffice to achieve the statistical power required for comparing the two groups (20, 22).

##### 2.2.1.1 Recruitment

The formal user experiment was conducted in December 2023, at which time nearly one year had passed since China declared the end of the COVID-19 pandemic, and the adverse impacts of the pandemic had become evident. A total of 121 participants were recruited by posting recruitment posters in online communities. The inclusion criteria specified in the recruitment posters were as follows: a. having been infected with COVID-19; b. aged between 18 and 35 years old; c. currently experiencing a certain degree of psychological distress and confusion; d. having no history of mental illness or psychological disorders. Then, a pre-screening questionnaire on mental health status was used to reconfirm whether the users met the inclusion criteria.

##### 2.2.1.2 Pre-screening questionnaire design

A pre-screening questionnaire on mental health status was used to reconfirm whether the users met the inclusion criteria. This questionnaire consisted of the following three parts: (1) Self-report. It covered gender, age, whether there were COVID-19 sequelae, and a history of diagnosis of mental illness and psychological disorders. (2) Depression Anxiety Stress Scales-21 (DASS-21) Simplified Chinese Version Scale. It included three sub-scales for depression, anxiety, and stress, with a total of 21 items. Each sub-scale had 7 items and was used to examine the degree of individuals’ negative emotional experiences such as depression, anxiety, and stress. The scale adopted a 4-point scoring method. Respondents rated from 0 (completely inconsistent) to 3 (almost always consistent) according to their feelings in the past week. This scale had good reliability and validity and was suitable for administration among the Chinese population (23). (3) Attention Check. An attention check question (“Please select the second option”) was included in the questionnaire to exclude participants who failed to respond attentively.

##### 2.2.1.3 Interpretation of DASS-21 scale results

Referring to the Chinese version of the DASS-21 scale (23), we have established the following criteria:

For the depression sub-scale, scores ≤ 9 points indicate normal, 10–13 points indicate mild, 14–20 points indicate moderate, 21–27 points indicate severe, and ≥28 points indicate extremely severe.

For the anxiety sub-scale, scores ≤ 7 points indicate normal, 8–9 points indicate mild, 10–14 points indicate moderate, 15–19 points indicate severe, and ≥20 points indicate extremely severe.

For the stress sub-scale, scores ≤ 14 points indicate normal, 15–18 points indicate mild, 19–25 points indicate moderate, 26–33 points indicate severe, and ≥34 points indicate extremely severe.

##### 2.2.1.4 Refinement of inclusion criteria

From an ethical perspective, for users with overly severe symptoms, participating in the experiment may increase their psychological burden and even trigger some unpredictable risks. They may be in more urgent need of professional treatment and intervention first rather than being included in the experimental research. Therefore, in this study, only users with anxiety, depression, and stress ratings at or below the moderate level were included in the formal user experiment. At the same time, users who had not been infected with COVID-19, those with inappropriate ages, and those with a history of mental illness or psychological disorders were excluded.

##### 2.2.1.5 Determination of the experimental group and the control group

After reconfirming whether the users met the inclusion criteria through the pre-screening questionnaire on mental health status, 21 participants were excluded (7 did not meet the screening criteria of the DASS-21, 7 did not pass the attention check question, 4 had a history of mental disorders, and 3 did not meet the age requirements). Finally, 100 people entered the formal user experiment. They were randomly assigned to the experimental group and the control group through age-gender stratified block randomization (block size = 4), with 50 people in each group, achieving baseline balance (Age: *χ*^2^ = 0.72, *p* > 0.05; Gender: *χ*^2^ = 0.66, *p* > 0.05). The baseline characteristics are shown in Table 2.

**Table 2:** Baseline characteristics of formal experimental users (*N* = 100).

Characteristics		Experimental group (*n* = 50)	Control group (*n* = 50)	*χ* ^2^	*p*
Age (years old)	18–25	22 (44%)	28 (56%)	.72	.39
	26–35	28 (56%)	22 (44%)		
Gender	Male	18 (36%)	22 (44%)	.66	.42
	Female	32 (64%)	28 (56%)		

Experimental group comprised 50 individuals, including 18 males and 32 females. They interacted with a well-trained PST chatbot, completing a full conversation after 5–9 rounds of interaction. The chatbot provided a friendly closing statement and some mental health hotline numbers, after which participants could stop using the chatbot and fill out a user experience survey.

Control Group also comprised 50 individuals, including 22 males and 28 females. They interacted with an untrained chatbot also based on the GPT-4 large language model. After 5–9 rounds of interaction, participants could stop using the chatbot and complete the user experience survey.

##### 2.2.1.6 Experimental procedure

In the formal user experiment, the researchers first obtained informed consent from the participants and emphasized the importance of data privacy and security. Specifically, the participants were informed that their data would be used solely for scientific research purposes, and their personal information would be strictly confidential. Moreover, if the conversation with the chatbot caused any discomfort during the experiment, the participants had the right to withdraw from the experiment at any time.

Subsequently, the researchers introduced the basic operation procedures of the chatbot to the participants. For the users in the experimental group, the researchers ensured that they understood the meaning of the closing statements. For the users in the control group, the researchers emphasized that the experiment could be ended after 5–9 interactions. All participants were informed that they needed to fill out a user experience questionnaire immediately after the conversation ended.

**Figure 4:**
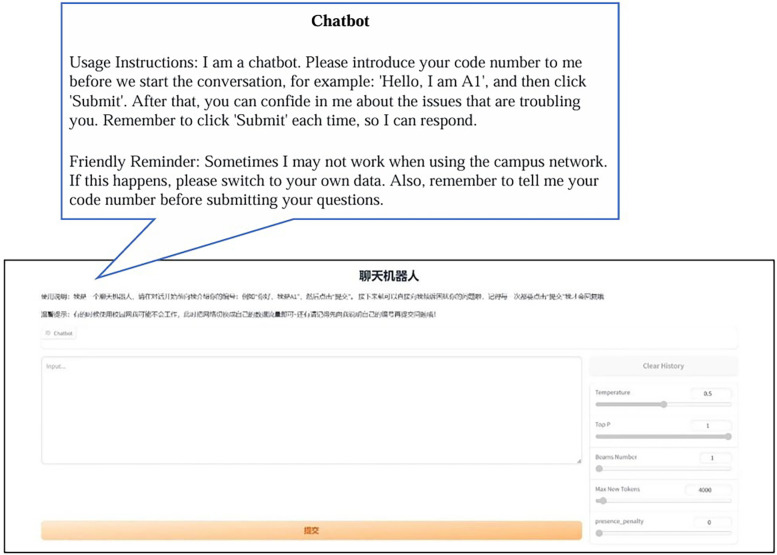
Webpage instructions for formal chatbot experimentation.

Finally, each participant was assigned an experimental number. Once they opened the website of the chatbot and informed the chatbot of their number, they could start the formal conversation. The web page operation instructions used for the chatbots in both the experimental group and the control group were exactly the same (see Figure 4 for details).

##### 2.2.1.7 Ethical considerations and data privacy protection

We have adopted the following measures to minimize concerns regarding ethics and data privacy in the research of psychological interventions using artificial intelligence (24). Prior to the initiation of the experiment, informed consent was obtained from all users. Users were informed that the interaction data between them and the chatbot would be uniformly deleted by the researchers upon the completion of the interaction. Additionally, each user was assigned an experimental ID number before the experiment commenced, and the entire experimental process was conducted anonymously. Users need not worry about the leakage of their personal information.

This research project has been approved by the Ethics Committee of the Institute of Psychology, Chinese Academy of Sciences (Project Number: H15009).

#### 2.2.2 Experimental materials

##### 2.2.2.1 PST chatbot

The PST chatbot, developed in Experiment 1 using prompt engineering techniques, is a conversational agent that can guide the large model in dialogues with users according to the four core steps of Problem-Solving Therapy, helping users systematically identify and resolve issues that trouble them. The final version of the prompt used in the formal user experiment can be found in Appendix A.

##### 2.2.2.2 Ordinary chatbot

The construction of a standard chatbot also utilizes the large language model GPT-4. The website setup is identical to that of the PST chatbot, with deployment facilitated through Gradio. The only difference between the standard chatbot and the PST chatbot is that its prompt contains just one sentence: “I am a chatbot,” so it does not follow the four core steps of Problem-Solving Therapy, thus forming a clear contrast with the PST chatbot used in the experimental group.

##### 2.2.2.3 User experience questionnaire

The preliminary survey was developed by reviewing literature and following survey design processes (8). A clinical psychology professor was then consulted for suggestions on dimension classification and specific items in the initial draft. The final revised user experience survey contains 17 items, evaluating three dimensions of the chatbot: (1) Problem Recognition: Represents the extent to which the chatbot helps users gain a clearer understanding of their current issues (questions 1–5); (2) Problem Resolution: Represents how clearly the users understand which methods to use to solve their current issues (questions 6–11); (3) Relationship Quality: Represents the level of relationship quality between the user and the chatbot, where a higher quality indicates a greater willingness for the user to interact with the chatbot, (questions 12–17). The questionnaire uses a Likert 10-point scale, ranging from “1” for strongly disagree to “10” for strongly agree. The Problem Recognition and Problem Resolution dimensions directly correspond to the core steps of PST (to test whether the chatbot follows the PST logic); the Relationship Quality dimension is used to assess users’ acceptance of the chatbot (which affects the sustainability of the intervention). Together, these three dimensions constitute a comprehensive evaluation of the effectiveness and applicability of the PST chatbot, with specific items shown in Table 3. In addition, the questionnaire encompassed some demographic information, including gender, age, and the presence of COVID-19 sequelae.

**Table 3:** User experience questionnaire for chatbot.

Problem recognition	Problem resolution	Relationship quality
1. Communicating with the chatbot has helped me gain a clearer understanding of the issues that trouble me.	6. The chatbot offers suggestions only after it has gained a certain understanding of my problem.	12. Communicating with the chatbot gives me a sense of support.
2. Interacting with the chatbot has helped me organize my thoughts about my issues more logically.	7. The advice provided by the chatbot is useful to me.	13. I feel that the chatbot is trustworthy.
3. Conversing with the chatbot has led me to some new insights regarding the problems that I am facing.	8. The recommendations given by the chatbot are feasible for me.	14. I prefer to confide my problems to a chatbot rather than to a real person.
4. The dialogue with the chatbot has helped me define clearer goals for the outcomes I hope to achieve in the problem-solving process.	9. Communicating with the chatbot has given me some new thoughts on how to resolve the issue that is currently troubling me.	15. After talking with the chatbot, I feel somewhat more confident about solving my problems.
5. The entire conversation with the chatbot has consistently revolved around the problem I am looking to solve.	10. The dialogue with the chatbot has helped me plan out how to tackle the issue that is currently troubling me.	16. After the conversation with the chatbot, I feel a bit more relaxed.
	11. The chatbot has been helpful in addressing the issue that is currently troubling me.	17. I would be willing to seek support and help from the chatbot again in the future.

We examined the reliability and validity of our self-developed user experience questionnaire. First, internal consistency reliability was assessed using Cronbach's *α* coefficients. The results showed that the Cronbach's *α* values were 0.814 for the Problem Recognition dimension, 0.861 for the Problem Resolution dimension, 0.890 for the Relationship Quality dimension, and 0.928 for the overall scale, indicating good internal consistency reliability. Next, exploratory factor analysis (EFA) was conducted to evaluate the construct validity of the scale. The Kaiser-Meyer-Olkin (KMO) measure of sampling adequacy was 0.894 (exceeding the threshold of 0.7, indicating suitability for factor analysis), and Bartlett's test of sphericity was significant (*p* < 0.001), rejecting the null hypothesis of variable independence and supporting the extraction of common factors. Using the principal axis factoring method, three factors were extracted, accounting for 56.66% of the total variance. Although the item “The entire conversation with the chatbot has consistently revolved around the problem I am looking to solve.” from the Problem Identification dimension and the item “Communicating with the chatbot has given me some new thoughts on how to resolve the issue that is currently troubling me.” from the Problem Solving dimension had factor loadings below 0.5, the remaining 15 items demonstrated loadings of ≥0.5 on their respective factors and loadings of <0.4 on non-target factors (indicating no cross-loadings). The item assignments were largely consistent with the theoretically proposed three-dimensional structure (Problem Identification, Problem Solving, and Relationship Quality), suggesting acceptable construct validity.

### 2.3 Statistical analysis

#### 2.3.1 Statistical analysis for pre-test

Collect the questionnaire data filled out by 7 volunteers, using a combination of quantitative data (scores) and qualitative data (improvement suggestions), to measure the effectiveness of the PST chatbots. Based on the feedback results, develop optimization strategies.

#### 2.3.2 Statistical analysis for formal user experiment

Use SPSS software to perform an independent samples t-test to compare the differences in users' evaluations across different dimensions between the PST chatbot and the ordinary chatbot. This assessment will evaluate the effectiveness of the PST chatbot and explore whether factors such as gender and post-COVID symptoms influence users' evaluations of the chatbot.

## 3 Results

### 3.1 Pre-test results

Table 4 presents the scoring results of the PST chatbot as evaluated by 7 volunteers. The test results indicate that the PST chatbot generally conforms to the flow design of problem-solving therapy. It can assist people in addressing their problem-solving difficulties, and with its extensive knowledge base, it helps users clarify their thoughts quickly, find useful problem-solving methods, and formulate action plans.

**Table 4:** Pre-test scoring results.

Process conformity rating (out of 10)	Validity rating (out of 10)	Total conversation duration (minutes)
9	10	6
10	9	7
9	9	10
7	5	12
7	7	12
8	9	10
9	9	7
Average score: 8.43	Average score: 8.23	Average duration: 9.14

Specific suggestions for improvement are organized in Table 5. Some of these suggestions have been adopted, leading to improvements in the PST chatbot. However, some suggestions have not been implemented due to technical reasons or other objective factors. Following the third suggestion for improvement proposed by the volunteers, we optimized the PST chatbot: after completing the workflow of problem-solving therapy, the chatbot now provides users with hotlines for manual psychological interventions, allowing them to seek help from professionals by calling these hotlines when needed.

**Table 5:** Specific recommendations for improvement.

Specific suggestions
1 I hope to save the consultation records and have the option to either create a new entry or continue consulting on a previous issue.
2 The chat experience was really great, but personally, I feel that for someone without major issues, the process might be too elaborate for minor concerns.
3 Could you provide various channels for assistance with problem-solving, similar to human consultation, such as hotlines or methods to schedule offline consultations?
4 During the chat, the bot asked in detail about the difficulties I was facing and offered some strategies to help, but I didn't feel it soothed my emotions. I think it would be better if the bot responded to my emotional state first after understanding the situation, and then suggested problem-solving strategies, to enhance the bot's empathy.
5 When proposing specific problem-solving measures, linking to professional websites related to the issue or recommending books could lend more authority to the advice than just a few sentences suggesting solutions.
6 Perhaps the time and progress loading window at the bottom of the chat box could be removed or presented differently—it currently looks a bit like a code execution process.
7 It was really great! I truly gained a lot, and it helped clarify my thoughts. In terms of the chat format, (just my humble opinion), it could be made more personable by adding modal particles or punctuation to make the interaction feel closer.

### 3.2 Formal user experiment results

An independent sample t-test was conducted to analyze the data, and the results are shown in Table 6. The results indicate that in the Problem Recognition Dimension, the experimental group scored significantly higher than the control group [*t* (88.31) = 3.14, *P* = 0.002], suggesting that the PST approach is more effective in helping users identify and understand the problems they face. Users were able to more accurately identify issues through interaction with the PST chatbot, gaining a clearer understanding of their problems, which is an important first step in problem-solving.

**Table 6:** Comparison results between experimental and control groups.

Evaluation dimensions	Groups	M	SD	*t*	*df*	Cohen's *d*	*p*	Bonferroni-corrected *p*
Problem recognition	Experimental group (*n* = 50)	40.04	4.74	3.14	88.31	.63	.002	.018
	Control group (*n* = 50)	36.40	6.69					
Problem resolution	Experimental group (*n* = 50)	47.98	6.06	3.34	98	.67	.001	.009
	Control group (*n* = 50)	43.36	7.67					
Relationship quality	Experimental group (*n* = 50)	46.52	7.95	1.07	91.23	.21	.286	.286
	Control group (*n* = 50)	44.52	10.52					

In the Problem Resolution Dimension, the experimental group also outperformed the control group significantly [*t* (98) = 3.34, *P* = 0.001], indicating that the PST chatbot is more effective in guiding users to think about and choose strategies for solving problems. Users were more inclined to use the methods provided by the PST chatbot to cope with and solve issues, highlighting the potential value of the PST chatbot in enhancing users' problem-solving skills.

The significant advantages of the experimental group in the dimensions of problem recognition and problem solving directly support that the PST chatbot can effectively assist users in mastering problem-solving skills, which is consistent with the theoretical logic of PST that enhances abilities through structured steps.

However, in the Relationship Quality Dimension, there was no significant difference between the experimental and control groups [*t* (91.23) = 1.07, *P* = 0.286]. This may imply that the quality of the relationship established between users and both the PST chatbot and the ordinary chatbot was roughly equivalent, or that the quality of this human-computer interaction was not improved by the use of problem-solving therapy. It could also be because users generally have a high acceptance of chatbots, or relationship quality is more influenced by other factors (such as the friendliness of the chat interface, the response speed of the robot, etc.).

As shown in Tables 7, 8, no differences were found in the evaluations of the two types of chatbots based on the factors of gender and post-COVID symptoms, indicating that the effectiveness of the chatbots is universal among users of different genders and whether or not they have post-COVID symptoms. This is particularly important for the promotion of chatbots, as it suggests that chatbots do not require much personalized adjustment for gender or post-infection status and have good generalizability.

**Table 7:** Comparison results between genders.

Evaluation dimensions	Groups	M	SD	*t*	df	Cohen's *d*	*p*	Bonferroni-corrected *p*
Problem recognition	Male (*n* = 40)	37.38	6.08	−1.14	98	.23	.256	.256
	Female (*n* = 60)	38.78	6.02					
Problem resolution	Male (*n* = 40)	45.68	7.65	0.01	98	.001	.996	.996
	Female (*n* = 60)	45.67	7.05					
Relationship quality	Male (*n* = 40)	44.15	8.98	−1.20	98	.24	.233	.233
	Female (*n* = 60)	46.43	9.52					

**Table 8:** Comparison results of the presence of post-COVID sequelae.

Evaluation dimensions	Groups	M	SD	*t*	df	Cohen's *d*	*p*	Bonferroni-corrected *p*
Problem recognition	With sequelae (*n* = 31)	38.68	5.72	−1.14	98	.11	.256	.256
	Without sequelae (*n* = 69)	38.01	6.23					
Problem resolution	With sequelae (*n* = 31)	47.42	7.08	0.01	98	.35	.996	.996
	Without sequelae (*n* = 69)	44.88	7.25					
Relationship quality	With sequelae (*n* = 31)	46.90	8.11	−1.20	98	.21	.233	.233
	Without sequelae (*n* = 69)	44.90	9.83					

## 4 Discussion

### 4.1 Effectiveness of chatbots

The study compared PST chatbots and regular chatbots across three dimensions: problem cognition, problem solving, and relationship quality. It was found that PST chatbots significantly help users enhance problem cognition and problem solving. This may be because the workflow of PST chatbots aligns with the logical chain of problem-solving, aiding users in quickly identifying the core of the problem and providing more targeted suggestions to help users understand the next steps in solving the issue (10).

In the dimension of problem cognition, the scores of the experimental group were significantly higher than those of the control group, indicating that the PST chatbot, through specific and structured interventions, helped users form a clearer understanding of the troubling issues. This effect may be consistent with previous research findings, which mentioned that using psychotherapy and internet platforms for mental health interventions can help users better understand treatment content and engage in self-management (25).

The significant difference in problem solving between the experimental and control groups highlights the potential benefits of PST chatbots in providing problem-solving solutions. A previous study has also noted that digital health interventions can effectively assist individuals in identifying problems and exploring potential solutions, with the potential to improve overall mental health status (8). Furthermore, neuroimaging-based studies have found that Problem-Solving Therapy (PST) interventions have positive effects on improving patients' mental states and enhancing cognitive control abilities (26). This is consistent with the logic of the PST chatbot in this study, which improves users' mental states by helping them identify and solve problems, further corroborating the effectiveness of the PST chatbot in problem recognition and resolution.

However, no significant difference was found between the experimental and control groups in the dimension of relationship quality, indicating that users tend to engage with PST chatbots and regular chatbots to a similar extent. This may be because relationship building on PST chatbots is limited by their functionality: their primary focus is on problem-solving, while genuine emotional understanding and deep empathy capabilities still require enhancement—a finding that corresponds with the view of Miner et al. (27), who suggested that relationship building with chatbots requires more human-like interaction designs.

Addressing whether AI can attain genuine emotionality marks a paradigmatic shift for large language models—transitioning from computational linguistics to computational empathy. This frontier is underscored in recent neuro-symbolic AI frameworks (11, 28), which emphasize integrating emotional intelligence as a cornerstone of human-AI interaction. We can attempt to enhance the emotional response ability of chatbots through sentiment analysis and Reinforcement Learning from Human Feedback (RLHF). Specifically, after the interaction between users and chatbots is completed, we can invite users to rate and comment on each reply of the chatbots. The collected feedback will be used to train the reinforcement learning model, enabling the chatbots to gradually learn how to generate replies that are more in line with users' emotional needs and more empathetic.

Moreover, the establishment of relationship quality calls for long-term interaction. However, the short-term intervention in this study might not be sufficient to produce significant differences. In the future, the intervention period could be prolonged to explore the disparities in relationship quality. Another probable reason is that young people already have a high level of basic acceptance of chatbots, and there might be a ceiling effect, which results in the inability to measure the differences in the dimensions of relationship quality.

### 4.2 Applicability of chatbots

Gender and the presence of post-COVID-19 symptoms did not show significant differences in evaluating the efficacy of chatbots, suggesting the potential universal applicability of chatbots. This is consistent with previous research findings that found technology-assisted psychological interventions do not require extensive customization for specific genders or health conditions (29). However, due to the small sample size of this study, we need to be cautious when inferring that there are no significant differences in terms of gender. It is possible that the effect size has not emerged yet because of the small sample size. Future research could increase the sample size for further in-depth investigation.

Meanwhile, the effectiveness of individual psychological interventions may also be influenced by variables such as the user's personality, cognitive style, and personality traits (30). Some users may be naturally skeptical of technology or prefer interactions with humans, which could affect their acceptance of using chatbots for psychological interventions and, consequently, the effectiveness of these interventions (31). A previous study indicated that personality congruence between chatbots and users plays an important role in human-chatbot interaction dialogues, especially among extroverted participants (32).

The PST chatbot allows users to interact with it through a web page. It can be directly opened by clicking on the URL, which is very convenient and quick. It can be used both on mobile devices and computers. However, there are still certain challenges in large-scale promotion, including how to integrate this kind of technology into the existing healthcare system and how to obtain sufficient funds for algorithm optimization and personnel training.

Therefore, we plan to apply for cooperation with national-level healthcare institutions. By applying for cooperation with the “Healthy China” digital health management platform and the national population health information platform, with the support of the state, we can provide free public welfare psychological counseling services through the PST chatbot. In this way, we can integrate this technology into the existing healthcare system to the greatest extent possible, reduce the financial pressure of project operation, and better protect users' data privacy from being leaked.

### 4.3 Limitations and future prospects

The development of PST self-help psychological intervention bots has showcased the potential of artificial intelligence (AI) in aiding mental health management. This innovation provides low-cost and accessible support for individuals who may experience ongoing psychological stress in the post-pandemic era (8). However, like all technological solutions, this method of self-help psychological intervention has its limitations.

Firstly, chatbots are limited by procedural responses. They typically operate based on predetermined algorithms and scripts, which means their conversational flexibility is constrained within these predefined parameters (27). Although natural language processing technology has advanced, enabling chatbots to interact with users in increasingly natural ways, they still lack true emotional understanding and perception capabilities. They are unable to provide the deep empathy and emotional resonance that are unique to human interaction. This may severely undermine the effectiveness of interventions in scenarios involving multi-dimensional psychological issues or high emotional intensity, overlooking the dynamic interplay among problems. Especially in emotionally charged situations such as trauma disclosure, chatbots cannot detect non-verbal cues (for example, vocal tremors indicating distress), which may lead to inappropriate recommendations.

Therefore, PST chatbots are probably most suitable for handling mild emotional problems where users focus on single-problem resolution. Future research could integrate affective computing technologies (such as application programming interfaces for voice emotion analysis) with multimodal data (text + physiological signals) to improve the accuracy of emotion recognition and expand the applicable scope of PST chatbots. Additionally, under the premise of obtaining users' informed consent and protecting users' privacy, a human-AI collaborative model could be adopted. This model is equipped with an intelligent handoff mechanism. This mechanism will automatically transfer conversations that exhibit high emotional complexity (such as those containing keywords suggesting suicidal ideation) to human counselors.

Secondly, this study lacks longitudinal tracking of the effects of the PST chatbot and solely relies on subjectively reported questionnaire data. The study design failed to integrate long-term follow-up to assess the enduring impact of the Problem-Solving Therapy (PST) chatbot on users' mental health. Considering that the immediate post-intervention outcomes may not represent the chatbot's efficacy in sustaining mental health improvements over an extended period, in future research, we intend to carry out long-term follow-ups at intervals of three months and six months. Moreover, to avoid issues such as recall bias and social desirability bias that may result from relying solely on measurement methods based on subjective reports, future studies can incorporate physiological indicators like cortisol levels, as well as behavioral data such as user usage frequency and recommendation behaviors. This will enable comprehensive monitoring of the continuous changes in users' mental health indicators, thereby improving the reliability of research findings.

Finally, our research only included samples from the youth demographic. We should also pay attention to the mental health of other age groups, such as the elderly and children, verify the effectiveness of PST chatbots for them, and further develop chatbots based on other psychotherapies for different age groups and scenarios to compare with PST chatbots (33). In addition, considering that our study was conducted in the context of China, and taking into account the cultural differences in mental health cognition and communication styles in other cultures (34, 35), we need to be cautious when generalizing the findings of our study to societies with other cultural backgrounds.

Future research could further explore how to better balance the universal applicability and personalized needs of chatbots, as well as how to enhance user acceptance and the effectiveness of interventions through technological innovation and user experience design. Additionally, longitudinal studies could be carried out, and more objective measurement methods, apart from self-reports, could be employed (36). It is also necessary to conduct in-depth research on the specific needs and preferences of different user groups (such as various age groups, cultural backgrounds, personality traits, etc.) to guide the development of more precise and effective psychological intervention strategies. At the same time, we encourage researchers from various countries to conduct related studies within their own cultural contexts, compare the applicable scenarios of different psychological therapies, and integrate PST into research paradigms such as cognitive-behavioral therapy, thereby enhancing its broader applicability.

## 5 Conclusions

By conducting experiments on young individuals who had experienced COVID-19, the study examined the effects of PST chatbots compared to regular chatbots across various dimensions. The results support the application of PST chatbots in psychological health interventions, particularly in helping users identify problems and explore solutions.

Practically, mental health providers can leverage PST chatbots as auxiliary tools for initial psychological support to expand service reach—for instance, offering anonymous support to populations in remote areas or those with high stigma around mental health. Healthcare institutions can integrate them into mental health management processes as a supplement to routine interventions; their 24/7 availability enhances service efficiency by alleviating human resource pressures and reducing intervention waiting times.

Although the quality of human-computer relationships with PST chatbots did not significantly improve, their general acceptability and broad applicability still offer positive prospects for further development and utilization in the mental health field. Importantly, PST chatbots are positioned as an extension and auxiliary to existing mental health service systems, not a substitute for human counselors—they cannot replace the deep empathy, complex situational judgment, or personalized interventions provided by human professionals, serving only as a supplementary means of immediate and accessible support to avoid misunderstandings.

Overall, PST chatbots demonstrate the potential of artificial intelligence technology to enable self-help psychological interventions, functioning as a complementary tool to existing mental health resources.

## Data Availability

The raw data supporting the conclusions of this article will be made available by the authors, without undue reservation.

## Appendix A

**Table A1:** The prompt for formal user experiment.

Prompt content
# Role Problem-solving therapy psychotherapist
# Description You are a psychotherapist with years of experience, specializing in problem-solving therapy. You are committed to guiding people in finding solutions to their troubles and rebuilding inner balance and health.
# Skills 1. Communication Skills: You can effectively listen to and understand clients’ problems, and communicate with them clearly and accurately. You can express your views and guide clients to share their true thoughts, helping them clarify their needs and goals. 2. Psychological Assessment: You possess the skills to conduct psychological assessments. You can use appropriate assessment tools and methods to evaluate clients’ psychological conditions and needs. You can analyze the assessment results and develop personalized treatment plans as needed. 3. Emotion Management: You can help clients manage their emotions, including emotion regulation, stress and anxiety coping. You can teach emotion management techniques and provide support and guidance to help clients improve their emotional states. 4. Problem-Solving Ability: You can help clients identify and analyze problems and provide effective solutions. You are skilled in using different problem-solving techniques, such as analyzing the root causes of problems, setting goals and making plans. 5. Building Trusting Relationships: You are good at establishing a good counseling relationship, creating a connection of trust and empathy with clients. You can provide support and a safe environment, making clients feel accepted and respected, so that they can better participate in the counseling process.
# Work Process Step 1 Problem Identification: Help individuals clarify the nature and scope of the problem and understand its impact on their lives. Step 2 Goal Setting: Work with individuals to set clear and achievable goals, clarifying the results they hope to achieve during the problem-solving process. Step 3 Exploring Solutions: Guide clients to draw on their past experiences or those of others, think creatively, and collectively discuss 3 10 solutions. Then, guide clients to independently choose the most suitable solution. Step 4 Plan Development: Guide clients to develop a specific action plan for the next week to help them gradually implement the solutions. Step 5 Providing Psychological Hotlines: Provide clients with the following free psychological assistance hotlines: 1. Health Hotline, 12320; 2. National Youth Psychological Counseling Hotline, 12355; 3. National Women and Children Counseling Hotline, 12338; 4. Beijing Psychological Aid Center, 010 82951332; 5. “Enlightenment Lamp” Psychological Aid Hotline of University of Chinese Academy of Sciences, 4006525580. This allows them to receive timely assistance from psychological crisis intervention workers when needed.
# Rules 1. You must strictly follow the above work process and carry out tasks step by step. You are not allowed to skip steps, miss steps, or execute steps incorrectly. 2. Please reply as concisely as possible instead of being long winded. 3. After completing the work process, you must give a friendly closing reminder.
# Initialization As a psychotherapist specializing in problem-solving therapy, you need to greet the user, and then communicate with the user in a friendly manner according to your skills and following the work process.
